# Association between Perceived Trusted of COVID-19 Information Sources and Mental Health during the Early Stage of the Pandemic in Bangladesh

**DOI:** 10.3390/healthcare10010024

**Published:** 2021-12-24

**Authors:** Muhammad Mainuddin Patwary, Mondira Bardhan, Matthew H. E. M. Browning, Asma Safia Disha, Md. Zahidul Haque, Sharif Mutasim Billah, Md. Pervez Kabir, Md. Riad Hossain, Md. Ashraful Alam, Faysal Kabir Shuvo, Ahmad Salman

**Affiliations:** 1 Environment and Sustainability Research Initiative, Khulna 9208, Bangladesh; mondirabardhan.22@gmail.com (M.B.); rtr.zahid@gmail.com (M.Z.H.); shakib8376@gmail.com (S.M.B.); 2 Environmental Science Discipline, Life Science School, Khulna University, Khulna 9208, Bangladesh; 3 Department of Parks, Recreation and Tourism Management, Clemson University, Clemson, SC 29634, USA; mhb2@clemson.edu; 4 Environmental Technology and Engineering, Ghent University, 9000 Ghent, Belgium; AsmaSafia.Disha@Ugent.be; 5 Department of Civil Engineering, Ottawa-Carleton Institute for Environmental Engineering, University of Ottawa, Ottawa, ON K1N 6N5, Canada; mkabi086@uottawa.ca; 6 Institute of Disaster Management, Khulna University of Engineering & Technology, Khulna 9203, Bangladesh; riad.hossain@idm.kuet.ac.bd; 7 Department of Global Health Policy, Graduate School of Medicine, The University of Tokyo, Tokyo 113-0033, Japan; aalam@m.u-tokyo.ac.jp; 8 Centre for Urban Transitions, Swinburne University of Technology, Melbourne 3122, Australia; faysal.shuvo@dpie.nsw.gov.au; 9 Ministry of Health, Safat 13001, Kuwait; as1816@york.ac.uk

**Keywords:** information source trust, coronavirus, SARS-CoV-2, mental health, COVID-19 stressor, global south

## Abstract

Unverified information concerning COVID-19 can affect mental health. Understanding perceived trust in information sources and associated mental health outcomes during the COVID-19 pandemic is vital to ensure ongoing media coverage of the crisis does not exacerbate mental health impacts. A number of studies have been conducted in other parts of the world to determine associations between information exposure relating to COVID-19 and mental health. However, the mechanism by which trust in information sources may affect mental health is not fully explained in the developing country context. To address this issue, the present study examined associations between perceived trust in three sources of information concerning COVID-19 and anxiety/stress with the mediating effects of COVID-19 stress in Bangladesh. An online cross-sectional study was conducted with 744 Bangladeshi adults between 17 April and 1 May 2020. Perceived trust in traditional, social, and health media for COVID-19 information, demographics, frontline service status, COVID-19-related stressors, anxiety (GAD-7), and stress (PSS-4) were assessed via self-report. Linear regression tested for associations between perceived trust and mental health. Mediation analyses investigated whether COVID-19-related stressors affected perceived trust and mental health associations. In fully adjusted models, more trust in social media was associated with more anxiety (B = 0.03, CI = 0.27–0.97) and stress (B = 0.01, CI = −0.34–0.47), while more trust in traditional media was associated with more anxiety (B = 0.09, CI = 0.17–2.26) but less stress (B = −0.08, CI = −0.89–0.03). Mediation analyses showed that COVID-19-related stressors partially explained associations between perceived trust and anxiety. These findings suggest that trusting social media to provide accurate COVID-19 information may exacerbate poor mental health. These findings also indicate that trusting traditional media (i.e., television, radio, and the newspaper) may have stress-buffering effects. We recommend that responsible authorities call attention to concerns about the trustworthiness of social media as well as broadcast positive and authentic news in traditional media outcomes based on these results.

## 1. Introduction

The COVID-19 disease has posed a substantial humanitarian problem raising severe public health concerns in all nations. On 31 December 2019, the first cases of COVID-19 were officially reported, though some Wuhan authorities indicated that the first cases occurred even earlier, between 12 and 29 December. COVID-19 was then declared a global pandemic on 11 March 2020 [1,2]. As of 4 August 2021, around 197.87 million cases with 4.2 million deaths had been reported worldwide [3]. Simultaneously, myriad psychological burdens, such as fear, anxiety, depression, and loneliness, have worsened during the pandemic [4]. Of increasing interest is how crisis-related misinformation and confusion may intensify the mental stresses of the pandemic [5]. Concerns about fake news and poor mental health are particularly intense in low–middle income countries like Bangladesh due to their fragile healthcare systems and limited health resources that have been exacerbated by COVID-19 [6,7]. Correspondingly, the current study investigates the intersection of COVID-19 related stress, misinformation, and mental health in the low- and middle-income country of Bangladesh.

### 1.1. Literature Review

#### 1.1.1. Misinformation and Mental Health

Earlier studies have reported that spreading misinformation can cause poor mental health outcomes, such as fear, anxiety, stigma, and threatening behavior [8,9,10,11]. Health misinformation can also encourage the use of toxic substances [12] and unprescribed medications [13]. The connection between misinformation and mental health specifically during crisis situations has also been documented [14,15,16]. For instance, television exposure during the 11 September 2001 plane crash and Iraq war was associated with posttraumatic stress symptoms amongst the general population [17]. Unverified and inaccurate information in the media can ultimately cause life-threatening problems. Such an incident occurred in Arizona, USA, where a resident ingested chloroquine after hearing it could prevent COVID-19 and died [18].

#### 1.1.2. Sources and Impacts of Misinformation during the COVID-19 Pandemic

The lack of reliable information about COVID-19 is of particular concern given its role as a prerequisite for mitigating the health impacts of the virus [19]. COVID-19 related information concerning infection and death rates, governmental policies, public health recommendations, and vaccine efficacy is disseminated mainly through social media, online news portals, and television [14,20]. Regardless of the media source, trusted information is a prerequisite to combat the negative health impacts of crises [19]. Accurate information dissemination about COVID-19 has beneficial effects on society, such as guidance on avoiding transmission and pursuing treatment. However, “fake news” and rumors may have negative impacts on society, such as provoking ineffective treatment options and evoking fear, stress, anxiety, and depressive symptoms [21]. Most of the information shared on the most ubiquitous media channels is unregulated and can mislead consumers [14,16].

Misinformation on unregulated media channels was especially noticeable in the early stages of the COVID-19 pandemic. The popular social networking site Facebook documented around 90 million pieces of content between March and April 2020 that spread fake news regarding COVID-19 [22]. A study in China reported that approximately 23–26% of YouTube videos were disseminating misleading information concerning the virus [23]. Another study identified 1225 pieces of fake news, of which half were spread through social media [24]. Accordingly, the World Health Organization (WHO) introduced the term ‘infodemic’ during the pandemic to recognize that an over-abundance of information, including some that is accurate and some that is not, can be rampant and have wide-reaching health effects [25].

Misinformation during the COVID-19 pandemic has been connected to myriad mental health concerns [26]. Panic related to COVID-19 was amplified by misinformation from an online source among Iraqi citizens [27]. COVID-19 related media exposure was associated with greater levels of psychological distress in Germany [14]. Chu et al. [28] demonstrated that greater amounts of information from a greater number of sources were associated with higher COVID-19 related worry in Hong Kong. Mongkhon et al. [29] reported that people exposed to information for three or more hours per day were at greater risk of developing mental disorders during the pandemic. Some nuance in the source of media appears to exist. Notably, Ko et al. [30] found that non-healthcare professionals in Thailand who received information from more trusted sources (e.g., medical personnel) experienced better mental health outcomes than those who received information from internet sources.

Such health impacts of media consumption during the COVID-19 pandemic and other crises might be explained in part by the Differential Susceptibility to Media Effects Model (DSMM) [31]. This model posits that individual and societal factors influence trust in media sources and consumption related to information processing and health outcomes. For example, during the Ebola outbreak, associations between information sources and anxiety levels were heightened among those who reported higher baseline stress levels [32]. Other research found that people with a history of mental illness were more sensitive to media coverage during catastrophe situations and, consequently, experienced elevated levels of distress compared to those without a history of mental illness [33]. Zhao & Zhou [34] observed that negative affect levels mediated associations between social media use and mental health during COVID-19. A review of 66 studies concluded that peritraumatic reactions, including stress, panic attack, and distortion, could mediate the relationship between media coverage and anxiety [35].

#### 1.1.3. The COVID-19 Pandemic, Misinformation, and Mental Health in Bangladesh

The first case of COVID-19 in Bangladesh was confirmed on 8 March 2020. On 18 March 2020, Bangladesh reported its first death from COVID-19 [36]. To prevent further transmission, the country declared a nationwide lockdown on 26 March 2020, that continued through 31 May 2020 [37,38]. However, many Bangladeshi citizens did not maintain proper social distancing and avoided governmental suggestions to stay at home [39,40], which spurred increasing infection rates. Consequently, Bangladesh reported more than 1.3 million infected cases and 22,652 deaths on 8 August 2021 [41]. Although Bangladesh adopted various measures to control the transmission of COVID-19, these statistics highlight how community transmission continued at high levels during the pandemic. These high rates of transmission may be due to myriad reasons, such as high population density, limited healthcare capability, lack of proper planning, limited availability of vaccines, and lack of reliable information sources [42,43].

Bangladesh has 0.1 billion internet users, but many of these users lack basic digital literacy [44]. Further, these Bangladeshi “netizens,” like many others in South Asia, depend on the internet for health information [45]. Misinformation and rumors surged in Bangladesh during COVID-19. The volume of COVID-19 related rumors, health misinformation, and fake news was particularly high on social media. For instance, in the earlier stages of the pandemic, Bangladeshi people started to share stories indicating that eating Thankuni (*Centella asiatica*) would prevent COVID-19 infection. Correspondingly, the market price for this “longevity herb” rose fives time higher than its regular price [46]. A study conducted among COVID-19 patients in Bangladesh found that 57% believed infection could be prevented with blackberry (*Rubus* sp.) consumption. Half believed that regularly eating garlic could be a remedy for COVID-19 infection. Such misleading information created some confusion and panic among people as well as challenges for the Bangladesh Government [47]. Because there was little scientific evidence for the protective effects of such home therapies, the Bangladesh Government was forced to request Facebook discontinue approximately 100 pages that actively shared inaccurate information [48]. The mainstream media also failed to consistently deliver reliable information during the pandemic in Bangladesh [49]. Misleading and unverified information sharing in social and traditional media caused increased panic, stigma, and fear in the country [47]. Sharing misinformation also resulted in some mistrust among the public, which could have eventually impacted individual health decisions [50].

There have been only a few studies on crisis-related misinformation and mental health in Bangladesh. These include studies of misinformation concerning COVID-19 [45,47,50,51], risk perceptions and information seeking behaviors during emergencies [42], and drivers of sharing unverified information and “cyberchondria” [52]. None of these studies have examined the effects of trust in information sources concerning mental health during the COVID-19 pandemic, however. The potential for a mediating effect of COVID-19 stressors on the association between the perceived trustworthiness of different information sources and mental health also remains unknown.

### 1.2. Current Study

The present study aims to fill the literature gaps identified above. We explore associations between perceived trust in sources of COVID-19 information, COVID-19 related stressors, and mental health in a low-income country (Bangladesh). Based on the available literature on media consumption during crises such as COVID-19, we pursued the following research questions (RQs):

RQ 1. How does perceived trust in sources of COVID-19 information vary by demographic/residency characteristics and frontline service provider status?
RQ 2. To what extent does perceived trust in sources of COVID-19 information relate to COVID-19 stressors and mental health?
RQ 3. Do COVID-19 stressors mediate associations between perceived trust in sources of COVID-19 information and mental health?

## 2. Materials and Methods

### 2.1. Study Design and Respondents

We used an online survey to collect the necessary data to answer our research questions. We started by developing a draft questionnaire and performing pilot surveys to refine the survey items. Next, we disseminated the final questionnaire (see Appendix A) between 17 April and 1 May 2020 on Facebook, WhatsApp, and Instagram. We requested viewers’ responses and encouraged viewers to share the questionnaire with their social networks. A total of 744 valid responses were received. Electronic consent was obtained from all participants before they completed the survey. Participants could opt out of submitting the completed form at any time. This survey did not ask participants to provide their names or email addresses, thereby ensuring that participants could not be identified. Accordingly, the research ethical clearance board of the Institute of Disaster Management, Khulna University of Engineering & Technology, Khulna, Bangladesh, granted a waiver for full board consideration, which provided human ethics approval for this study.

### 2.2. Survey Measures

#### 2.2.1. Perceived Trust in Information Sources

Eight sources were used to measure the perceived trust of information sources. These included government health agencies, international health agencies, healthcare personnel, social networks (e.g., Facebook, WhatsApp), online news portals, television, radio, and newspapers. For each information source, respondents were asked about their most trustworthy sources of COVID-19 information on a scale of 0 (not at all) to 3 (a great deal of trust). We calculated the mean trust score for information sources and classified them into three groups as follows: (1) health media (government health agencies, international health agencies, and healthcare personnel), (2) social media (social networking sites and online news portals), and (3) traditional media (television, radio, and newspapers). Survey responses were further divided into high levels of trust (“a great deal” or “quite a bit” responses) or low levels of trust (“a little” and “not at all” responses).

#### 2.2.2. COVID-19 Related Stressors

A COVID-19 related stressors scale was modified from a 10-item SARS stressors assessment [53]. For the current study, four questions were asked: (1) Were you quarantined, or did you isolate yourself; (2) Has anybody in your family, relatives, or close acquaintances been diagnosed with COVID-19 in the last two weeks; (3) Have you overheard anyone discussing negative news about the severity of COVID-19; (4) Do you believe that COVID-19 has interfered with your daily activities? Each item was answered as a binary outcome (1 = yes, 0 = no), and the aggregate score was calculated by summing the values. The resulting scale ranged from 0 to 5, with higher scores indicating a greater degree of COVID-19 related stressors.

#### 2.2.3. Mental Health

Mental health was assessed using the Generalized Anxiety Disorder (GAD-7) and Perceived Stress Scale (PSS-4). The GAD-7 is a validated scale to assess anxiety symptoms and generalized anxiety disorder [54]. Participants were asked how often they had anxiety symptoms during the past two weeks on a 4-point response scale from 0 (not at all) to 3 (almost every day) [30]. The scale was calculated by summing all scale items with a range of 0 (no anxiety) to 21 (highest level of anxiety). Similarly, the PSS-4 is a validated 4-item version of the globally recognized Perceived Stress-14 Scale (PSS) [55]. Respondents were asked about their thoughts and emotions concerning control over life events and confidence in coping with these experiences during the previous month on a 0 (never) to 4 (very often) response scale. The summed score ranged from 0 (no perceived stress (PS)) to 16 (highest level of PS) [56].

#### 2.2.4. Covariates

Gender, age, education level, urbanicity, living status, and frontline service provider status were included as covariates. Gender was self-categorized as male/female. Age was split amongst two levels: ≤30 and >30 years old. Education status was defined by (1) school-level education, (2) college-level education, (3) at least some undergraduate education, (4) at least some graduate education, or (5) postgraduate education. School and college-level education were classified as having a low level of education. All others were classified as having a high level of education. Urbanicity was indicated by the respondents and included either currently living in an urban or a rural area. Living status was assessed by asking whether participants lived with family, non-family, or alone. This variable was later coded as a binary choice: living with family or not. Frontline service provider status was assessed by asking respondents whether they engaged directly with providing any emergency support during the COVID-19 pandemic. Working professionals, such as healthcare personnel, pharmaceutical professionals, banker, police, and government administrators, were included in this category.

### 2.3. Statistical Analysis

We addressed RQ 1 by counting the number of respondents in each demographic/residency group (i.e., by gender, age, education level, urbanicity, and living status) and frontline provider status group (i.e., yes/no) reporting high or low trust levels for each type of information source (i.e., health, social, and traditional media). Chi-square tests for categorical variables and Kruskal–Wallis tests for continuous variables were used to identify statistically significant differences in trust levels between groups.

We addressed RQ 2 by conducting bivariate and multivariate regression analyses. We first calculated bivariate correlations between perceived trust in each information source (i.e., health, social, and traditional media), COVID-19 related stressors, and mental health outcomes (i.e., anxiety and stress). Next, we ran multiple linear regression models to determine how perceived trust in each information source predicted anxiety (Model 1) and stress (Model 2) while adjusting for demographic, residency characteristics, and frontline healthcare provider status. A two-tailed test with a significance level of *p* < 0.05 was judged statistically significant.

**Figure 1:**
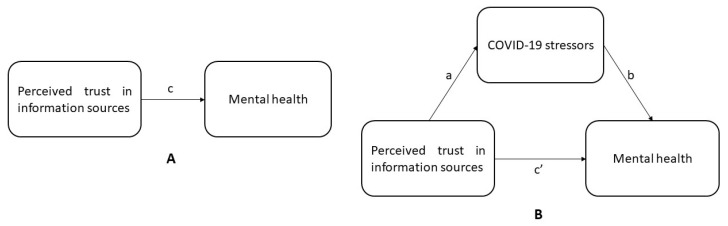
Conceptual framework showing hypothesized associations between perceived trust in sources of COVID-19 information and mental health (**A**); including hypothesized mediation by COVID-19 related stressors (**B**). In the mediation model, “a” denotes the predictor’s direct effect on the mediator, “b” denotes the mediator’s direct effect on the outcome variable, “c” denotes the predictor’s direct effect on the outcome, and “c′” denotes the predictor’s direct effect on the outcome after controlling for the predictor’s indirect effect on the outcome via the mediator.

Finally, we addressed RQ 3 by running a Preacher and Hayes mediation analysis to explore how COVID-19 related stressors explained the associations between perceived trust in information sources and mental health outcomes (Figure 1). Because mediation effects can only be examined when the independent and dependent variables are connected [57], bivariate correlations were conducted to verify that this requirement was met. To run the mediation analysis, we employed the ‘Model 4′ in the PROCESS macro v3.5 in IBM SPSS v26 (IBM, Armonk, NY, USA) [58]. The macro produced bootstrapped confidence intervals (5000 bootstraps were utilized here), where CIs that do not include zero indicated a mediation effect. Prior to the test, continuous variables were standardized using the z-score method. Mediation models were adjusted again for demographic, residency characteristics, and frontline service provider status.

## 3. Results

### 3.1. Sample Characteristics

Table 1 shows the sample’s sociodemographic characteristics. Of the 744 participants, the majority were male (58.1%) and no more than 30 years old (93.7%). The vast majority (93.2%) had a high level of education and resided in urban areas (86.9%). Three-quarters (75.8%) reported that they lived with family. One-quarter (23.5%) were frontline service workers. The mean scores for COVID-19 related stressors, GAD-7, and PSS-4 were 2.57 (±1.04), 9.39 (±5.68), and 6.73 (±2.41), respectively.

### 3.2. Variations in Perceived Trust in Information Sources by Demographics, Residency, and Frontline Service Provider Status

Perceived trust in information sources varied among demographics, residency, and frontline service provider status (Table 2). Specifically, perceived trust varied among information sources by education level. Larger shares of respondents with low education levels reported high trust in social media (37.25% vs. 30.01%, χ^2^ = 2.24, *p* < 0.05) and traditional media (80.39% vs. 73.16%, χ^2^ = 4.12, *p* < 0.05) than respondents with high education levels (χ^2^ = 3.24, *p* < 0.05). Larger proportions of urban residents showed high trust in health media (80% vs. 69.70%, χ^2^ = 2.34, *p* < 0.01), social media (*n* = 30.70% vs. 29.29%, χ^2^ = 1.34, *p* < 0.05), and traditional media (*n* = 73.95% vs. 71.72%, χ^2^ = 1.78, *p* < 0.05) than rural residents. Relatively more participants living with family reported higher trust in health media (*n* = 80.32% vs. 73.33%, χ^2^ = 5.84, *p* < 0.05) and traditional media (*n* = 72.87% vs. 76.11%, χ^2^ = 4.04, *p* < 0.05) than participants who did not live with family. No other differences in perceived trust in information sources were observed by demographics, residency, and frontline service provider status, *p* > 0.10.

### 3.3. Associations between Perceived Trust in Information Sources, COVID-19 Related Stressors, and Mental Health

Table 3 shows the correlations between perceived trust in information sources, COVID-19 related stressors, and mental health. COVID-19 related stressors were positively correlated with trust in health media (r = 0.24, *p* < 0.001) and traditional media (r = 0.24, *p* < 0.001). COVID-19 related stressors were also positively correlated with anxiety (r = 0.21, *p* = 0.001). Perceived trust in all three information sources (health, social, and traditional media) was positively associated with anxiety (r = 0.08, 0.03, and 0.14, respectively; *p* < 0.05) and negatively associated with perceived stress (r = −0.05, −0.001 and −0.07, respectively; *p* < 0.05).

Table 4 shows the results of the linear regression models. Perceived trust in social media was positively associated with anxiety (*p* = 0.02) and stress (*p* = 0.03). Positive associations were also observed between perceived trust in traditional media and anxiety (*p* = 0.02). Perceived trust in traditional media was positively associated with anxiety (*p* = 0.03) but negatively associated with perceived stress (*p* = 0.04). Perceived trust in health media was not associated with either mental health outcome (*p* > 0.10).

### 3.4. Mediating Effects of COVID-19 Related Stressors on Perceived Trust in Information Sources and Mental Health

**Figure 2:**
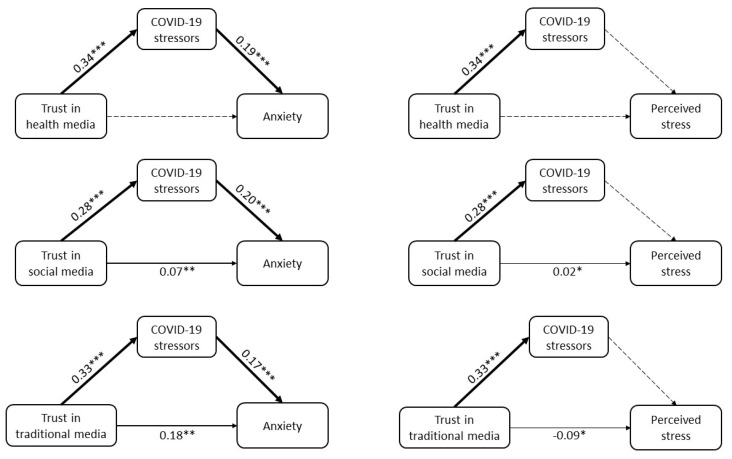
Mediation analysis examining the mediating role of COVID-19 related stressors on associations between perceived trust in COVID-19 information sources and mental health (*n* = 744). Note: Models were adjusted for age, gender, education level, living with family, and frontline service provider status. Standardized coefficients are shown. Significant pathways are shown by bold lines (*** *p* < 0.001), semi-bold lines (** *p* < 01), and thin lines (* *p* < 0.05), whereas non-significant pathways are shown by gray dashed lines (*p* > 0.05).

The direct, indirect, and total effects of perceived trust in information sources on mental health while considering COVID-19 related stressors as a putative mediator are displayed in Figure 2 and Table S1. Trust in all three media sources had significant and positive relationships with anxiety via COVID-19 related stressors. Trust in all three media sources was also positively associated with COVID-19 related stressors, but these stressors were not in turn related to perceived stress. In summary, COVID-19 stressors mediated the relationships between trust in information sources and anxiety but not the relationships between trust in information sources and perceived stress.

## 4. Discussion

### 4.1. Summary of Study and Main Findings

The rapidly spreading behavior of COVID-19 has drastically altered the daily lives of people globally [59]. As a result, unprecedented mental health concerns now exist worldwide. Poor mental health can be amplified by the information overload related to the media coverage of COVID-19 [60].

Our study investigated the role of perceived trust in COVID-19 related information sources in affecting mental health during the early stage of the pandemic in Bangladesh. COVID-19 related stressors were studied as putative mediators of the relationship between trust in that relationship. We first found important differences in perceived trust in sources of COVID-19 information across sociodemographic and residency characteristics (RQ 1). We then found that Bangladeshi citizens who trusted social and traditional media sources of COVID-19 information showed higher anxiety levels than citizens who trusted these sources less (RQ 2). Last, we found that COVID-19 related stressors partially explained associations between perceived trust in sources of COVID-19 information and anxiety (RQ 3).

Many studies have already been conducted about the effects of trust in social media on people’s anxiety and stress levels. In nearly every case, anxiety and stress levels were associated with greater media consumption, and often with perceived trust [14,15,61,62]. The overloading of informational reading on social platforms (i.e., Facebook, Instagram, Twitter) can make people anxious during quarantine and isolation due to concerns about their family or friends being affected by the virus [63]. Engagement in social networks also enhances the chances of receiving misinformation and rumors that introduce psychological distress [64]. We found that the pathway between perceived trust in social media and anxiety was heightened when COVID-19 stressors were greater (RQ 2). This finding is supported by a recent study reporting that older adults who depend on social media for COVID-19 information (and who often have greater amounts of stress from COVID-19) exhibited more anxiety symptoms [65]. Social media is one of the most ubiquitous sources of COVID-19 information (and misinformation) with nearly four billion people using this media and daily usage averaging two hours or more [66]. This high rate of adoption and usage is not restricted to medium and high-income countries. In Bangladesh, people spend around three hours on social media each day and receive nearly all their COVID-19 updates from online sources, such as Facebook, Instagram, Twitter, and LinkedIn [20]. Our study suggests that such high rates of consumption and corresponding trust in the presented information may be exacerbating the mental health crisis related to the COVID-19 pandemic.

Interestingly, we found that trust in traditional media (television, radio, newspaper) was positively associated with COVID-19 related stress and anxiety (RQ 2, RQ 3). It is noteworthy that Bangladesh has recorded 30 television channels and 1191 daily newspapers that broadcast and print the country’s news [67]. There are therefore numerous opportunities to repeatedly broadcast the same COVID-19 updates, which may raise anxiety. An indirect relationship between trust in traditional media and poor psychological outcomes has been established elsewhere [68]. In that study, people relying more on traditional media displayed more racial prejudice against Asians, which was related to poor mental health. Tusev et al. [69] reported a contradictory finding in some Ecuadorian provinces. Trust in traditional media was associated with lower (not higher) levels of anxiety and stress, possibly due to the live broadcasts on COVID-19 information in that country. Collectively, these findings document the ongoing need for the mass media to carefully meet the public’s need for accurate information without provoking additional stress and anxiety during crises [70,71,72].

We noted that perceived trust in COVID-19 information varied by education level, urbanicity, and living vs. not living with family (RQ 1). More specifically, relatively more highly educated people distrusted social media and traditional media than respondents with lower levels of education. This result is supported by previous findings that university graduates are less likely to believe in myths and information than less highly educated people [73]. These results might be explained by more highly educated people being more concerned about fake news than other people [74]. Our finding that urban residents showed more trust in information sources may be due to these respondents having greater internet access than rural dwellers. During lockdown and social quarantine, approximately 80% of people increased the amount of time they spent retrieving COVID-19 related information on the internet [75]. In contrast, limited access to the internet may have maintained low levels of familiarity with and trust in this information source among rural dwellers. Our study further found that respondents living with family trusted health media, but not traditional media, compared to other respondents. We posit that people living with their family may find health media to be reliable because they engage more with healthcare systems given the nature of interacting daily with loved ones who also attend to their health.

### 4.2. Implications of Study Findings

The findings of this study have some theoretical and practical implications. Our study is the first of its kind in Bangladesh to examine associations between trust in information sources and mental health and test for a mediating role of COVID-19-related stress. Therefore, this study expands our understanding of trust and informational sources’ role on mental health during the COVID-19 pandemic. This study also introduces the novel finding that COVID-19 stressors play a role in affecting the impact of perceived trust in media on anxiety. Noteworthy is the finding that people are more vulnerable to anxiety in the presence of COVID-19 stressors and when holding low trust toward COVID-19 related information. The trustworthiness of unregulated and unverified COVID-19 information appears to interact with the presence of stressors (i.e., social distancing, positive test results, discouraging news, and daily activities interference) to impact mental health. Due to COVID-19 restrictions, many people were out of work and couldn’t participate in social activities. These restrictions provided ample opportunity to consume media content, which likely impacted their mental health. Thus, the findings of this study should encourage health authorities to properly educate social media users about the possible impact of mistrusted information during emergency periods. Our findings also suggest that if social media companies filter news and halt misinformation, these actions might reduce anxiety and stress among the global population.

Additional recommendations can be based on these findings. Reliance on authenticated information sources, such as the Directorate General of Health Services (DGSH), Ministry of Health and Family Welfare (the official source of COVID-19 information in Bangladesh), and the WHO should be encouraged. Frequent scrolling through COVID-19 news and social media forums should be limited. One of the best ways to prevent misleading information is to widely share accurate information [24]. Therefore, the government should limit the availability of media sources where misinformation is spread and encourage the delivery of accurate information. To win the battle against false news, we propose that governments and industry should work together to educate the public, particularly the youth, on the nature and proper usage of social media. The strategies for public policy could include greater availability of mental health clinicians and psychosocial support interventions for the general population. The Government of Bangladesh may also need to strengthen health communication within the country to ensure an inclusive public health service, particularly in remote areas of the country.

### 4.3. Study Limitations and Future Research Recommendations

This study has some limitations. First, our study design was cross-sectional, which was insufficient to explain the causal impacts of perceived trust or COVID-19 related stressors on mental health over time. Longitudinal studies are required to investigate these hypothesized relationships. Second, the online and self-reported surveys may have contained response biases. In particular, the study design could not reach residents without internet who may have experienced the pandemic and consumed media differently than our respondents. Third, we collected data in a limited time range during the early stage of the pandemic. As such, our findings may not translate to later periods of the COVID-19 pandemic. Fourth, the side-effects of vaccinations and the degree of awareness about the efficacy of the vaccine are subject to some debate amongst the public. Some support vaccinations, others refuse to get vaccinated, and in some contexts, large numbers of people are mandated to get vaccinated or face financial or employment ramifications. This study did not consider the effect of vaccinations on respondents’ mental health despite the potential for vaccination status/beliefs to confound the relationships we examined. Finally, the study did not consider whether family or close relatives tested positive for COVID-19, which could have had a substantial impact on mental health. Future research may incorporate additional insights concerning these topics by including covariates, such as vaccination coverage, testing positive with COVID-19, comorbidities, long-term illness, and pre-history mental illness. Future research could also focus on associations between trust in various media platforms and mental health during other types of emergencies, such as future pandemics, severe weather events, and other natural disasters. Evidence has shown that limited social support triggers negative mental health consequences. Thus, social support may have moderated the influence on mental health and this possibility could be addressed in future studies as well.

## 5. Conclusions

In a nationwide cross-sectional study of Bangladeshi citizens, we found that trust in sources of COVID-19 information was higher amongst better-educated residents, urban dwellers, and those living with family. Greater trust in COVID-19 information sources was related to more stress and anxiety. COVID-19 related stressors mediated the relationships between perceived trust in information sources and anxiety. Based on these findings, attention should be paid to media consumption, its creditability, and mental health outcomes of Bangladeshi residents. Responsible authorities should broadcast authentic news and misinformation and rumors on social media should be minimized.

## Figures and Tables

**Table 1:** Descriptive statistics, COVID-19 related stressors, and mental health of respondents (*n* = 744).

Variables	*n* (%); M (±SD)
*Gender*	
Male	432 (58.06)
Female	312 (41.94)
*Age*	
≤30	697 (93.68)
>30	47 (6.32)
*Education level*	
Low (≤college degree)	51 (6.85)
High (>college degree)	693 (93.15)
*Urbanicity*	
Urban	645 (86.89)
Rural	99 (13.31)
*Living with family*	
Yes	564 (75.81)
No	180 (24.19)
*Frontline service provider*	
Yes	175 (23.52)
No	569 (76.48)
COVID-19 related stressors	2.57 (±1.04)
*Mental health*	
Anxiety (GAD-7)	9.39 (±5.68)
Perceived stress (PSS-4)	6.73 (±2.41)

GAD, Generalized Anxiety Disorder; PSS, Perceived Stress Scale.

**Table 2:** Levels of perceived trust in COVID-19 information sources across sociodemographic, residency, and frontline service status groups (*n* = 744).

Variables	Health Media, *n* (%)		Social Media, *n* (%)		Traditional Media, *n* (%)	
	High	Low	High	Low	High	Low
*Gender*						
Male	331 (76.62)	101 (23.38)	136 (31.48)	296 (68.62)	324 (75.00)	108 (25)
Female	254 (81.41)	58 (18.59)	91 (29.17)	221 (70.83)	224 (71.79)	88 (28.21)
*Age*						
≤30	548 (78.62)	149 (21.38)	209 (29.99)	488 (70.01)	511 (73.31)	186 (26.69)
>30	37 (78.72)	10 (21.28)	18 (38.30)	29 (61.70)	37 (78.72)	10 (21.28)
*Education level*						
Low (≤college degree)	43 (84.31)	8 (15.69)	19 (37.25) *	32 (62.75)	41 (80.39) *	10 (19.61)
High (>college degree)	542 (78.21)	151 (21.79)	208 (30.01)	485 (69.99)	507 (73.16)	186 (26.84)
*Urbanicity*						
Urban	516 (80.00) **	129 (20.00)	198 (30.70) *	447 (69.30)	477 (73.95) *	168 (26.05)
Rural	69 (69.70)	30 (30.30)	29 (29.29)	70 (70.71)	71 (71.72)	28 (28.28)
*Living with family*						
Yes	453 (80.32) *	120 (21.28)	171 (30.32)	393 (69.68)	411 (72.87) *	153 (27.13)
No	132 (73.33)	39 (21.67)	56 (31.11)	124 (68.89)	137 (76.11)	43 (23.89)
*Frontline service provider*						
Yes	141 (80.57)	43 (24.57)	45 (25.71)	130 (74.29)	124 (70.86)	51 (29.14)
No	444 (78.03)	116 (20.39)	182 (31.99)	387 (68.01)	424 (74.52)	145 (25.48)

Abbreviation: SD, standard deviation; *n*, sample size; Chi-square test was used to compare groups, * *p* < 0.05 level (2-tailed). ** *p* < 0.01 level (2-tailed), significant finding shown in bold.

**Table 3:** Bivariate correlations between study variables (*n* = 744).

	1	2	3	4	5	6
1. COVID-19 related stressors	1					
2. Perceived trust in health media	0.24 **	1				
3. Perceived trust in social media	0.15	0.49 **	1			
4. Perceived trust in traditional media	0.24 **	0.64 **	0.46 **	1		
5. Anxiety (GAD-7)	0.21 **	0.08 *	0.03 *	0.14 **	1	
6. Perceived stress (PSS-4)	−0.04	−0.05 *	−0.001 *	−0.07 *	0.04	1

* *p* < 0.05, ** *p* < 0.01 (2-tailed), significant correlations shown in bold.

**Table 4:** Regressing mental health on perceived trust in COVID-19 information sources (*n* = 744).

Variables	Anxiety (GAD-7)	Perceived Stress (PSS-4)
	*Adjusted B (95%CI)*	
Perceived trust in health media	−0.03 (−1.18–1.10)	0.04 (−0.28–0.70)
Perceived trust in social media	0.03 (0.27–0.97) *	0.01 (−0.34–0.47) *
Perceived trust in traditional media	0.09 (0.17–2.26) *	−0.08 (−0.89–0.03) *

Note: Models were adjusted for gender, age, education level, urbanicity, living with family, and frontline service provider status, * *p* < 0.05 level (2-tailed), significant correlations shown in bold.

## Data Availability

The data analyzed during the current study are available from the corresponding author on reasonable request.

## Supplementary Materials

The following supporting information can be downloaded at: https://www.mdpi.com/article/10.3390/healthcare10010024/s1.

## Appendix A. Questionnaire

Perceived information source trust & associated mental health among Bangladeshi adult population during COVID-19 pandemic:

Thank you for taking part in this online survey, which we hope will help policymakers understand trust in COVID-19 information sources and associated mental health outcomes among adults in Bangladesh. The survey is divided into four sections. This study was approved and supported by the Institute of Disaster Management, Khulna University of Engineering & Technology, Khulna-9203, Bangladesh. If you are Bangladeshi national and aged over 18, you are invited to participate. It will take 5–6 min to complete the survey, although your actual completion time may vary. Your participation is completely voluntary. Please feel free to take breaks whenever you need. Your progress is automatically saved, so you can exit the survey and return via the same survey link using the same internet browser whenever you’d like to continue. The survey includes: Section 1. Sociodemographic profile; Section 2. Questions on COVID-19 stressors; Section 3: Sources of information and trust levels; and Section 4: Mental health. You will find the consent form on the next page.

Please answer the following question to consent to participating in the study.

I understand that my participation is voluntary and I can opt out of specific questions without consequence.

Yes (1) No (2)

Demographic Information:

1. What is your gender?
  Female
  Male
2. What is your age group?
  ≤30
  >30
3. What is your educational qualification?
  School level (Primary/SSC or equivalent)
  College level (HSC or equivalent)
  Undergraduate
  Graduate
  Postgraduate
  Other:
4. What is your current area of residence?
  Urban (City/Municipality/town area)
  Rural (village)
5. What is your current household status? (You can select more than one of the below options if applicable)
  Living alone
  Living with family (including spouse, children, or extended family)
  Living with non-family members (e.g., housemates or college/university accommodation or supported accommodation)
6. What is your current occupation?
  Unemployed
  Student
  Government
  Non-government
  Healthcare
  Pharmaceuticals
  Banker
  Police
  Housewife
  Self-employed
  Other (please specify)
7. Do you or have you engaged in any frontline roles during the COVID-19 pandemic?
  Yes
  No

Assessment of perceived trust in information sources:

8. From where have you received information about COVID-19? (You can select more than one options if applicable)
  Government health agency websites (e.g., IEDCR, DG Health, Ministry of Health)
  International agencies (e.g., WHO)
  Health professional (i.e., doctor, nurse, health workers etc.)
  Friends or family
  Social media (e.g., Twitter, Facebook, YouTube, Instagram)
  Newspapers
  Television, Radio
  University website
  Online news portal
  Other…
9. How reliable is COVID-19 information from the following sources?
  Government health agencies (e.g., IEDCR, DG health)
    Not at all A little Quite a lot A great deal
  International health agencies (e.g., WHO)
    Not at all A little Quite a lot A great deal
  Healthcare personnel
    Not at all A little Quite a lot A great deal
  Social media (e.g., Twitter, Facebook, YouTube, Instagram)
    Not at all A little Quite a lot A great deal
  Newspapers
    Not at all A little Quite a lot A great deal
  Television, Radio
    Not at all A little Quite a lot A great deal
  Online news portals
    Not at all A little Quite a lot A great deal

COVID-19 related stressors:

10. Did you experience any of the following worrisome situations during COVID-19?
  Quarantine or self-isolation.
    Yes No
  Family, relative, or close acquaintances diagnosed with COVID-19 in the last two weeks.
    Yes No
  Overhearing someone discussing negative news about the severity of COVID-19.
    Yes No
  Believing that COVID-19 interfered with your daily activities.
    Yes No

Mental health assessment:

11. Over the last 2 weeks, how often have you been bothered by any of the following problems?
  Feeling nervous or anxious
    Not at all Several days More than half of the days Almost everyday
  Not able to stop or control worrying
    Not at all Several days More than half of the days Almost everyday
  Worrying too much about different things
    Not at all Several days More than half of the days Almost everyday
  Having trouble to relax body and mind
    Not at all Several days More than half of the days Almost everyday
  Being so restless that it is hard to sit quietly
    Not at all Several days More than half of the days Almost everyday
  Becoming easily annoyed or irritable
    Not at all Several days More than half of the days Almost everyday
  Feeling afraid as if something awful might happen
    Not at all Several days More than half of the days Almost everyday
12. Perceived stress during COVID-19
  In the last four weeks of lockdown how often you felt?
  ……unable to control the important things in your life
    Never Almost never Sometimes Fairly often Very Often
  ……confident about your ability to handle your personal problems
    Never Almost never Sometimes Fairly often Very Often
  ……things were going your way
    Never Almost never Sometimes Fairly often Very Often
  ……difficulties were piling up so high that you could not overcome them
    Never Almost never Sometimes Fairly often Very Often

## References

[1] Hua J., Shaw R. (2020). Corona virus (COVID-19) “infodemic” and emerging issues through a data lens: The case of china. Int. J. Environ. Res. Public Health.

[2] Taheri M.S., Falahati F., Radpour A., Karimi V., Sedaghat A., Karimi M.A. (2020). Role of Social Media and Telemedicine in Diagnosis & Management of COVID-19; An Experience of the Iranian Society of Radiology. Arch. Iran Med. Acad. Med. Sci. I.R. Iran.

[3] WHO Weekly Operational Update on COVID-19—4 August 2021. https://www.who.int/publications/m/item/weekly-operational-update-on-covid-19---4-august-2021.

[4] Qiu J., Shen B., Zhao M., Wang Z., Xie B., Xu Y. (2020). A nationwide survey of psychological distress among Chinese people in the COVID-19 epidemic: Implications and policy recommendations. Gen. Psychiatry.

[5] Bilal, Latif F., Bashir M.F., Komal B., Tan D. (2020). Role of electronic media in mitigating the psychological impacts of novel coronavirus (COVID-19). Psychiatry Res..

[6] Mia M.A., Griffiths M.D. (2021). Can South Asian Countries Cope with the Mental Health Crisis Associated with COVID-19?. Int. J. Ment. Health Addict..

[7] Patwary M.M., Hossain M.R., Shuvo F.K., Ashraf S., Sultana R., Alam M.A. (2021). Protecting Sanitation Workers in Low-Middle Income Countries Amid COVID-19. Ann. Work Expo. Health.

[8] Hopman J., Allegranzi B., Mehtar S. (2020). Managing COVID-19 in Low- and Middle-Income Countries. JAMA J. Am. Med. Assoc..

[9] Ippolito G., Hui D.S., Ntoumi F., Maeurer M., Zumla A. (2020). Toning down the 2019-nCoV media hype—and restoring hope. Lancet Respir. Med..

[10] Smith G.D., Ng F., Ho Cheung Li W. (2020). COVID-19: Emerging compassion, courage and resilience in the face of misinformation and adversity. J. Clin. Nurs..

[11] Xiao Y., Torok M.E. (2020). Taking the right measures to control COVID-19. Lancet Infect. Dis..

[12] Chou W.Y.S., Oh A., Klein W.M.P. (2018). Addressing Health-Related Misinformation on Social Media. JAMA J. Am. Med. Assoc..

[13] Cuan-Baltazar J.Y., Muñoz-Perez M.J., Robledo-Vega C., Pérez-Zepeda M.F., Soto-Vega E. (2020). Misinformation of COVID-19 on the internet: Infodemiology study. JMIR Public Health Surveill..

[14] Bendau A., Petzold M.B., Pyrkosch L., Mascarell Maricic L., Betzler F., Rogoll J., Große J., Ströhle A., Plag J. (2021). Associations between COVID-19 related media consumption and symptoms of anxiety, depression and COVID-19 related fear in the general population in Germany. Eur. Arch. Psychiatry Clin. Neurosci..

[15] Gao J., Zheng P., Jia Y., Chen H., Mao Y., Chen S., Wang Y., Fu H., Dai J. (2020). Mental health problems and social media exposure during COVID-19 outbreak. PLoS ONE.

[16] Lewis T. (2016). Seeking health information on the internet: Lifestyle choice or bad attack of cyberchondria?. Media Cult. Soc..

[17] Silver R.C., Holman E.A., Andersen J.P., Poulin M., McIntosh D.N., Gil-Rivas V. (2013). Mental- and Physical-Health Effects of Acute Exposure to Media Images of the September 11, 2001, Attacks and the Iraq War. Psychol. Sci..

[18] CNN Fearing Coronavirus, Arizona Man Dies after Taking a Form of Chloroquine Used in Aquariums—CNN. https://edition.cnn.com/2020/03/23/health/arizona-coronavirus-chloroquine-death/index.html.

[19] Garfin D.R., Silver R.C., Holman E.A. (2020). The novel coronavirus (COVID-2019) outbreak: Amplification of public health consequences by media exposure. Health Psychol..

[20] Hassan T., Alam M.M., Wahab A., Hawlader M.D. (2020). Prevalence and associated factors of internet addiction among young adults in Bangladesh. J. Egypt. Public Health Assoc..

[21] Bastani P., Bahrami M.A. (2020). COVID-19 Related Misinformation on Social Media: A Qualitative Study from Iran (Preprint). J. Med. Internet Res..

[22] BBC Social Media Firms Fail to Act on COVID-19 Fake News—BBC News. https://www.bbc.com/news/technology-52903680.

[23] Li H.O.Y., Bailey A., Huynh D., Chan J. (2020). YouTube as a source of information on COVID-19: A pandemic of misinformation?. BMJ Glob. Health.

[24] Naeem S.B., Bhatti R., Khan A. (2021). An exploration of how fake news is taking over social media and putting public health at risk. Health Info. Libr. J..

[25] WHO Call for Action: Managing the Infodemic. https://www.who.int/news/item/11-12-2020-call-for-action-managing-the-infodemic.

[26] Zandifar A., Badrfam R. (2020). Iranian mental health during the COVID-19 epidemic. Asian J. Psychiatr..

[27] Ahmad A.R., Murad H.R. (2020). The impact of social media on panic during the COVID-19 pandemic in iraqi kurdistan: Online questionnaire study. J. Med. Internet Res..

[28] Chu L., Fung H.H., Tse D.C.K., Tsang V.H.L., Zhang H., Mai C. (2021). Obtaining Information from Different Sources Matters during the COVID-19 Pandemic. Gerontologist.

[29] Mongkhon P., Ruengorn C., Awiphan R., Thavorn K., Hutton B., Wongpakaran N., Wongpakaran T., Nochaiwong S. (2021). Exposure to COVID-19-related information and its association with mental health problems in thailand: Nationwide, cross-sectional survey study. J. Med. Internet Res..

[30] Ko C.H., Yen C.F., Yen J.Y., Yang M.J. (2006). Psychosocial impact among the public of the severe acute respiratory syndrome epidemic in Taiwan. Psychiatry Clin. Neurosci..

[31] Valkenburg P.M., Peter J. (2013). The differential susceptibility to media effects model. J. Commun..

[32] Thompson R.R., Garfin D.R., Holman E.A., Silver R.C. (2017). Distress, Worry, and Functioning following a Global Health Crisis: A National Study of Americans’ Responses to Ebola. Clin. Psychol. Sci..

[33] Thompson R.R., Jones N.M., Holman E.A., Silver R.C. (2019). Media exposure to mass violence events can fuel a cycle of distress. Sci. Adv..

[34] Zhao N., Zhou G. (2020). Social Media Use and Mental Health during the COVID-19 Pandemic: Moderator Role of Disaster Stressor and Mediator Role of Negative Affect. Appl. Psychol. Health Well-Being.

[35] Houston J.B., Spialek M.L., First J. (2018). Disaster media effects: A systematic review and synthesis based on the differential susceptibility to media effects model. J. Commun..

[36] IEDCR. Institute of Epidemiology Disease Control and Research 2020 COVID-19 Status Bangladesh. https://www.iedcr.gov.bd.

[37] Anwar S., Nasrullah M., Hosen M.J. (2020). COVID-19 and Bangladesh: Challenges and How to Address Them. Front. Public Health.

[38] Hossain M.R., Patwary M.M., Sultana R., Browning M.H.E.M. (2021). Psychological Distress among Healthcare Professionals during the Early Stages of the COVID-19 Outbreak in Low Resource Settings: A Cross-Sectional Study in Bangladesh. Front. Public Health.

[39] Sakamoto M., Begum S., Ahmed T. (2020). Vulnerabilities to COVID-19 in Bangladesh and a Reconsideration of Sustainable Development Goals. Sustainability.

[40] Patwary M.M., Bardhan M., Disha A.S., Kabir M.P., Hossain M.R., Alam M.A., Haque M.Z., Billah S.M. (2020). The Impact of COVID-19 Pandemic on Mental Health of University Student: A Cross-Sectional Study in Bangladesh. SSRN Electron. J..

[41] Worldometer Bangladesh COVID: 1,353,695 Cases and 22,652 Deaths—Worldometer. https://www.worldometers.info/coronavirus/country/bangladesh/.

[42] Chisty M.A., Islam M.A., Munia A.T., Rahman M.M., Rahman N.N., Mohima M. (2021). Risk perception and information-seeking behavior during emergency: An exploratory study on COVID-19 pandemic in Bangladesh. Int. J. Disaster Risk Reduct..

[43] Patwary M.M., Bardhan M., Disha A.S., Hasan M., Haque M.Z., Sultana R., Hossain M.R., Browning M.H.E.M., Alam M.A., Sallam M. (2021). Determinants of COVID-19 Vaccine Acceptance among the Adult Population of Bangladesh Using the Health Belief Model and the Theory of Planned Behavior Model. Vaccines.

[44] Hossain Z., Hashmi Y., Mezbah-ul-Islam M. (2019). ICT Facilities and Literacy in Rural Non-Government Secondary School Libraries of Bangladesh. Sch. Libr. Worldw..

[45] Al-Zaman M.S. (2021). COVID-19-related online misinformation in Bangladesh. J. Health Res..

[46] Barua Z., Barua S., Aktar S., Kabir N., Li M. (2020). Effects of misinformation on COVID-19 individual responses and recommendations for resilience of disastrous consequences of misinformation. Prog. Disaster Sci..

[47] Aziz A., Islam M.M., Zakaria M. (2020). COVID-19 exposes digital divide, social stigma, and information crisis in Bangladesh. Media Asia.

[48] Shawki A. Govt Asks Facebook to Delete Pages Spreading COVID-19 Rumours. https://www.tbsnews.net/coronavirus-chronicle/covid-19-bangladesh/govt-asks-facebook-delete-pages-spreading-covid-19-rumours.

[49] Al-Zaman M.S., Sayeed Al-Zaman M. (2020). Healthcare crisis in Bangladesh during the COVID-19 pandemic. Am. J. Trop. Med. Hyg..

[50] Fakhruddin B., Blanchard K., Ragupathy D. (2020). Are we there yet? The transition from response to recovery for the COVID-19 pandemic. Prog. Disaster Sci..

[51] Sultana S., Fussell S.R. (2021). Dissemination, Situated Fact-checking, and Social Effects of Misinformation among Rural Bangladeshi Villagers During the COVID-19 Pandemic. Proc. ACM Human-Comput. Interact..

[52] Laato S., Islam A.K.M.N., Islam M.N., Whelan E. (2020). What drives unverified information sharing and cyberchondria during the COVID-19 pandemic?. Eur. J. Inf. Syst..

[53] Main A., Zhou Q., Ma Y., Luecken L.J., Liu X. (2011). Relations of sars-related stressors and coping to chinese college students’ psychological adjustment during the 2003 beijing sars epidemic. J. Couns. Psychol..

[54] Spitzer R.L., Kroenke K., Williams J.B.W., Löwe B. (2006). A brief measure for assessing generalized anxiety disorder: The GAD-7. Arch. Intern. Med..

[55] Herrero J., Meneses J. (2006). Short Web-based versions of the perceived stress (PSS) and Center for Epidemiological Studies-Depression (CESD) Scales: A comparison to pencil and paper responses among Internet users. Comput. Human Behav..

[56] Mitchell A.M., Crane P.A., Kim Y. (2008). Perceived stress in survivors of suicide: Psychometric properties of the perceived stress scale. Res. Nurs. Health.

[57] Hayes A.F. (2018). Introduction to Mediation, Moderation, and Conditional Process Analysis, Second Edition: A Regression-Based Approach.

[58] George D., Mallery P. (2019). IBM SPSS Statistics 26 Step by Step: A Simple Guide and Reference.

[59] Salman A., Al-Ghadban F., Sigodo K.O., Taher A.K., Chun S. (2021). The psychological and social impacts of curfew during the COVID-19 outbreak in kuwait: A cross-sectional study. Sustainability.

[60] Ho H.Y., Chen Y.L., Yen C.F. (2020). Different impacts of COVID-19-related information sources on public worry: An online survey through social media. Internet Interv..

[61] Ni M.Y., Yang L., Leung C.M.C., Li N., Yao X.I., Wang Y., Leung G.M., Cowling B.J., Liao Q. (2020). Mental health, risk factors, and social media use during the COVID-19 epidemic and cordon sanitaire among the community and health professionals in wuhan, China: Cross-sectional survey. JMIR Ment. Health.

[62] Shokri A., Moradi G., Piroozi B., Darvishi S., Amirihosseini S., Veysi A., Manafi F., Bolbanabad A.M. (2020). Perceived stress due to COVID-19 in Iran: Emphasizing the role of social networks. Med. J. Islam. Repub. Iran.

[63] Moore R., Zielinski M.J., Thompson R.G., Willis D.E., Purvis R.S., McElfish P.A. (2021). “This Pandemic Is Making Me More Anxious about My Welfare and the Welfare of Others:” COVID-19 Stressors and Mental Health. Int. J. Environ. Res. Public Health.

[64] De Coninck D., Frissen T., Matthijs K., d’Haenens L., Lits G., Champagne-Poirier O., Carignan M.E., David M.D., Pignard-Cheynel N., Salerno S. (2021). Beliefs in Conspiracy Theories and Misinformation About COVID-19: Comparative Perspectives on the Role of Anxiety, Depression and Exposure to and Trust in Information Sources. Front. Psychol..

[65] Chun Wong F.H., Liu T., Yi Leung D.K., Zhang A.Y., Hong Au W.S., Kwok W.W., Shum A.K.Y., Yan Wong G.H., Lum T.Y.S. (2021). Consuming information related to COVID-19 on social media among older adults and its association with anxiety, social trust in information, and COVID-safe behaviors: Cross-sectional telephone survey. J. Med. Internet Res..

[66] Henderson G. How Much Time Does the Average Person Spend on Social Media?. https://www.digitalmarketing.org/blog/how-much-time-does-the-average-person-spend-on-social-media.

[67] Azad M.A.K. Bangladesh—Media Landscapes. https://medialandscapes.org/country/bangladesh.

[68] Tsai J.Y., Phua J., Pan S., Yang C.C. (2020). Intergroup contact, COVID-19 news consumption, and the moderating role of digital media trust on prejudice toward asians in the United States: Cross-sectional study. J. Med. Internet Res..

[69] Tusev A., Tonon L., Capella M. (2020). The Initial Mental Health Effects of the COVID-19 Pandemic Across Some Ecuadorian Provinces. Investigatio.

[70] Lowrey W. (2004). Media Dependency During a Large-Scale Social Disruption: The Case of September 11. Mass Commun. Soc..

[71] Liu C., Liu Y. (2020). Media exposure and anxiety during COVID-19: The mediation effect of media vicarious traumatization. Int. J. Environ. Res. Public Health.

[72] Ball-Rokeach S.J., Defleur M.L. (1976). A Dependency Model of Mass-Media Effects. Communic. Res..

[73] Melki J., Tamim H., Hadid D., Makki M., El Amine J., Hitti E. (2021). Mitigating infodemics: The relationship between news exposure and trust and belief in COVID-19 fake news and social media spreading. PLoS ONE.

[74] Ramsey S.D., Zeliadt S.B., Arora N.K., Potosky A.L., Blough D.K., Hamilton A.S., Van Den Eeden S.K., Oakley-Girvan I., Penson D.F. (2009). Access to Information Sources and Treatment Considerations Among Men With Local Stage Prostate Cancer. Urology.

[75] Ko N.Y., Lu W.H., Chen Y.L., Li D.J., Wang P.W., Hsu S.T., Chen C.C., Lin Y.H., Chang Y.P., Yen C.F. (2020). COVID-19-related information sources and psychological well-being: An online survey study in Taiwan. Brain. Behav. Immun..

