# Supplementary material for: Association between Perceived Trusted of COVID-19 Information Sources and Mental Health during the Early Stage of the Pandemic in Bangladesh
Source: Healthcare (Basel). 2021 Dec 24;10(1):24. doi: 10.3390/healthcare10010024 (PMC8775621; doi:10.3390/healthcare10010024)
Supplement: Supplementary file 1 [file healthcare-10-00024-s001.zip › healthcare-1468883-supplementary.pdf]

healthcare

MDPI

Supplementary Table S1. Mediation analysis

**Pathways and effects**		**β**	**SE**	**95% CI**
Perceived trust in health media → COVID-19 stressors → anxiety				
	Direct	0.22	0.27	-0.31 to 0.75
	Indirect	0.36*	0.08	0.21 to 0.53
	Total	0.58*	0.26	0.060 to 1.107
Perceived trust in social media→ COVID-19 stressors → anxiety				
	Direct	0.01*	0.06	-0.11 to 0.14
	Indirect	0.05***	0.01	0.02 to 0.09
	Total	0.07	0.06	-0.05 to 0.19
Perceived trust in traditional media→ COVID-19 stressors → anxiety				
	Direct	0.12**	0.04	0.03 to 0.21
	Indirect	0.05***	0.01	0.02 to 0.08
	Total	0.17***	0.04	0.08 to 0.27
Perceived trust in health media→ COVID-19 stressors → perceived stress				
	Direct	-0.05	0.04	-0.15 to 0.03
	Indirect	-0.01	0.01	-0.03 to 0.01
	Total	-0.6	0.04	0.14 to -0.16
Perceived trust in social media→ COVID-19 stressors → perceived stress				
	Direct	0.01	0.06	-0.12 to 0.14
	Indirect	-0.01	0.01	-0.03 to 0.01
	Total	-0.01	0.06	-0.13 to 0.12
Perceived trust in traditional media→ COVID-19 stressors → perceived stress				
	Direct	-0.8	0.04	-0.18 to 0.01
	Indirect	-0.01	0.01	-0.03 to 0.01
	Total	-0.09*	0.04	-0.18 to -0.01

CI, Confidence Interval; SE, Standard Error;

*$p < .05$ level, **$p < .01$, ***$p < .001$ level (2-tailed).
